# Versatile Polycaprolactone-Based Drug Delivery System with Enhanced Cytocompatibility and Antibacterial Activity

**DOI:** 10.3390/jfb16050182

**Published:** 2025-05-15

**Authors:** Celine Guder, Anja Hofmann, Therese Schüler, Torsten Sterzenbach, Hans-Peter Wiesmann, Katrin Lorenz, Christian Hannig, Christian Reeps, Benjamin Kruppke

**Affiliations:** 1 Institute of Materials Science, Max Bergmann Center of Biomaterials, Technische Universität Dresden, 01069 Dresden, Germany; celine.guder@tu-dresden.de (C.G.); therese.schueler@mailbox.tu-dresden.de (T.S.); hans-peter.wiesmann@tu-dresden.de (H.-P.W.); 2 Division of Vascular and Endovascular Surgery, Department of Visceral, Thoracic and Vascular Surgery, Medical Faculty, University Hospital Carl Gustav Carus, Technische Universität Dresden, 01307 Dresden, Germany; anja.hofmann2@ukdd.de (A.H.); christian.reeps@ukdd.de (C.R.); 3 Policlinic of Operative Dentistry, Periodontology and Pediatric Dentristy, Medical Faculty, University Hospital Carl Gustav Carus, Technische Universität Dresden, 01307 Dresden, Germany; torsten.sterzenbach@ukdd.de (T.S.); katrin.lorenz@ukdd.de (K.L.); christian.hannig@ukdd.de (C.H.)

**Keywords:** degradable polyester, composite processing, drug release, smooth muscle cells, drug delivery system, antibiotics, antimicrobial, cytotoxicity

## Abstract

Common antibiotic therapies to treat bacterial infections are associated with systemic side effects and the development of resistance, directly connected to duration and dosage. Local drug delivery systems (DDSs) offer an alternative by localising antibiotics and thereby limiting their side effects while reducing the dosage necessary. A biodegradable polyester polycaprolactone (PCL)-based DDS was thus produced, containing various clinically relevant drugs. It was shown that the incorporation of four distinct antibiotic classes (amoxicillin, doxycycline, metronidazole and rifampicin), with very high mass fractions ranging up to 20 wt%, was feasible within the PCL matrix. This DDS showed the capacity for effective and sustained release. The release kinetics over 14 days were proven, showing a significant decrease in cytotoxicity with smooth muscle cells as well as an antibacterial effect on (1) aerobic, (2) anaerobic, (3) Gram-positive and (4) Gram-negative pathogens in vitro. The DDS demonstrated a markedly diminished cytotoxic impact owing to sustained release in comparison to pure antibiotics, while simultaneously maintaining their antibacterial efficacy. In conclusion, DDSs are a more tolerable form of antibiotics administration due to the hydrophobic PCL matrix causing a slower diffusion-controlled release, proven as a release mechanism via the Peppa–Sahlin model.

## 1. Introduction

Antimicrobial and antibiotic agents are the most frequently used drugs worldwide. The efficiency of antimicrobial therapy strongly depends on its capacity to achieve concentrations equal to or greater than the minimum inhibitory concentration (MIC) at the site of the infection. In addition, there may be differences in activity at certain sites in the body. In most cases, the serum concentrations do not reflect the concentrations at problem sites [1]. Adverse side effects include allergies, drug toxicity, cross-activity with other drugs or indirect effects on the commensal flora, e.g., the gut, oropharyngeal, skin and vaginal microflora [1,2]. Owing to the systemic side effects associated with prolonged treatment, the selection of antibiotic-resistant organisms and the high costs, duration of treatment and dosage used are of particular importance.

Drug delivery systems (DDSs) provide an alternative form of application that effectively avoids the disadvantages mentioned above [3]. DDSs offer the ability to release the drug in a targeted and controlled manner, sparing the rest of the body from the adverse effects associated with conventional delivery methods. DDSs are therefore employed in a diverse range of applications, including, e.g., periodontitis treatments utilising doxycycline-loaded hydrogel [4] and antibiotic-enriched bone cements in endoprosthetics [5]. Antibiotics-enriched DDSs have also been tested in vascular graft infection (VGI) [6]. VGI is a serious complication in prosthetic vascular surgery, with an incidence of 1–6% and mortality rates between 15 and 75%. Synthetic graft materials (e.g., polytetrafluoroethylene (PTFE) and polyethylene terephthalate (PET)) are, in general, prone to bacterial adhesion and therefore need an additional antibacterial treatment. In the clinical practise, synthetic vascular grafts are commonly silver-coated or rifampicin-impregnated for local treatment at the surgical site [7]. The latter means soaking the graft in a rifampicin solution during surgery. Despite the significant reduction in re-infection observed in studies, this method is not suitable for long-term therapy since the effect is only short-lived, owing to the rapid release of the antibiotic [7,8,9]. Furthermore, its burst release leads temporarily to cytotoxic concentrations that may damage the surrounding tissue and prevent cell adhesion, proliferation and ultimately tissue regeneration. This is a common side effect not to be tolerated in material/tissue contact especially in context of DDSs in vascular grafts and for periodontitis treatment with respect to gingiva preservation.

Particularly for vascular grafts, the body fluids that surround the implant are inadequately perfused and these fluids help bacteria to proliferate. In consequence, these bacteria trigger a graft infection and prevent optimal attachment to the surrounding tissue [10]. In fact, the graft material should promote a sufficient tissue incorporation promptly after implantation despite the known cytotoxicity of antibiotics [11]. The production of the extracellular matrix (ECM) by smooth muscle cells and fibroblasts is particularly important for vascular healing and must not be affected by the DDS [12,13].

Biodegradable polymers represent an alternative approach as a DDS, as they should be ingrown with new tissue and replaced by the ECM [14]. Additionally, the antibiotic should be provided from a degradable matrix to adjust its release kinetics and provide effective antibacterial concentrations. Ideally, the matrix material is degraded after the antibiotic release to not trigger detrimental effects.

A potential drug delivery matrix is polycaprolactone (PCL, [15,16,17]), which is a biocompatible, FDA approved, semi-crystalline polyester that has already been extensively characterised [18]. As a thermoplastic with a low melting temperature (approximately 60 °C), PCL offers straightforward options for processing via melt-based techniques, such as mould casting and 3D printing, and allows the incorporation of heat-sensitive additives. Here, PCL shows a loading capacity of up to 71 wt% [19]. A high proportion of antibiotics is essential for a DDS with a medium- to long-term effect. PCL offers the advantage that it can be completely degraded in the body by hydrolysis, with subsequent resorption [18]. Owing to their relatively long degradation times (>12 months), PCL-based DDSs are suitable for long-term applications.

As elucidated by Liu, Chen et al. [20], the combination of hydrophobic materials, such as PCL, with poorly water-soluble antibiotics (e.g., rifampicin) is particularly suitable for this purpose. Based on this knowledge, PCL-based DDSs do allow a high antibiotic loading for sustained drug release while preventing cytotoxicity and maintaining antibacterial activity.

Although the analysis of PCL as a carrier material for DDSs has already been extensively documented in the literature, this study supplements this knowledge by showing how PCL can be processed with comparatively high amounts of active agents, confirming its antibacterial efficacy (after exposure to the process temperature), owing to its sustained antibiotic release, and also demonstrating that the composite material leads to improved biocompatibility upon contact with tissue cells. This is the necessary starting point for making PCL usable as a DDS for use as a drug-releasing scaffold, filament, fleece or similar. The processing conditions, such as the temperature exposure of the material and the antibiotics, the active ingredient content in the matrix and the release of the active ingredient from the carrier (PCL), as well as the comparability of clinically highly relevant antibiotics, should make it possible to develop specific DDSs that are adapted to the therapeutic goal through their processing form.

## 2. Materials and Methods

### 2.1. Materials

Amoxicillin sodium, doxycycline hyclate and metronidazole were obtained from Hycultec (Beutelsbach, Germany), rifampicin from Roth (Karlsruhe, Germany), polycaprolactone (MW = 23,500 g/mol) from Thermo Scientific Chemicals (Waltham, MA, USA) and Dulbecco’s phosphate-buffered saline (PBS) as well as acetonitrile were obtained from Sigma-Aldrich (Taufkirchen, Germany).

### 2.2. Production of PCL-Based DDS

The active ingredients employed were amoxicillin (AMX), doxycycline (DXC), metronidazole (MNZ) and rifampicin (RIF). To incorporate these into the polymer matrix, the PCL granules were melted at 90 °C, the corresponding amount of antibiotic (0.02/0.2/2/20 wt%) was added and the mixture was homogenised manually. The aforementioned mass was subsequently processed into granules by means of spreading it on an anti-adhesive film, cooling it and subsequently cutting it. The granulate was weighed (along with the pure PCL for the reference samples) and moulded into cylindrical test specimens (m = 71 mg, d = 5 mm, h = 3.5 mm). To assess their homogeneity, light microscope images of the sample cross-sections were taken using a VHX 5000 digital microscope (Keyence, Osaka, Japan).

### 2.3. In Vitro Antibiotic Release Profile

The release profile of the antibiotics from the PCL was determined by a quasi-static 14 d incubation experiment. Prior to the commencement of the experiment, the dry masses of the samples (n = 3) were determined, and then incubated in 1 mL PBS at 37 °C and 5% CO_2_ for the duration of the experiment. At specified time points (4 h, 1 d, 3 d, 7 d, 10 d, 14 d), the medium was completely removed for further analysis and replaced with fresh PBS. At the conclusion of the experiment, the samples were dried at 37 °C and the masses determined.

#### 2.3.1. Determination of the Active Substance Concentration Using UV/Vis

The antibiotic concentrations in the eluates of the incubation test were determined using ultraviolet–visible (UV/Vis) spectroscopy. A volume of 50 µL was pipetted into UV-Star^®^ Microplates (Greiner Bio-One) for analysis using an infinite 200Pro device (TECAN, Männedorf, Switzerland). Firstly, a spectrum of the low-concentration solutions of the four antibiotics in PBS was recorded over the wavelength range of 230–700 nm (Figure S1). The same procedure was then repeated for PCL dissolved in acetonitrile in a quartz cuvette (Hellma, Jena, Germany). The characteristic absorbance maxima determined (Table 1), were thus employed for subsequent measurements. A calibration series was then created for all the antibiotics in PBS, with the absorbance values of the pure PBS subtracted as blanks. In each case, a mean regression line (n = 3, R^2^ ≥ 0.998) was determined through the zero point, which was used to convert the measured absorbance values of the samples into antibiotic concentrations (Figure S2). The mean absorbance from three technical replicates was employed for the determination. Should the absorbance of a sample exceed the value of 2, it was diluted with PBS and the dilution factor was added to the concentration.

The eluates of the pure PCL were also analysed at the characteristic wavelengths of the antibiotics (derived from their spectra) in order to calculate an antibiotic equivalent. Similarly, the reference spectrum of the pure PCL was employed to assess the impact of the material on the antibiotic concentration determination.

#### 2.3.2. Mathematical Analysis of the Drug Release Kinetics

The basis for calculating the drug release rates were the antibiotic concentration curves over time determined using UV/Vis. Various typical mathematical models were used, including zero order Equation (1), first order Equation (2), Higuchi Equation (3) and Peppas–Sahlin Equation (4).

(1) $$\mathrm{Zero}\;\mathrm{Order}\;Q_{t}=K\times t$$

(2) $$\mathrm{First}\;\mathrm{Order}\;ln\;Q_{r}=-K\times t+Q_{0}$$

(3) $$\mathrm{Higuchi}\;Q_{t}=K\times t^{0.5}$$

(4) $$\mathrm{Peppas}-\mathrm{Sahlin}\;Q=K_{1}\times t^{m}+K_{2}\times t^{2m}=F+R$$

where $Q_{t}$ is the amount of antibiotic released over time t, $Q_{0}$ is amount of antibiotic incorporated, $Q_{r}$ is the amount of antibiotic remaining at time t, n and m are exponents of release, and $K,\;K_{1}$ and $K_{2}$ are release constants. The Peppas–Sahlin model is the only model that considers the Fickian diffusional contribution and relaxational contribution separately by the two terms F (diffusion) and R (relaxation).

### 2.4. Agar Diffusion Tests to Determine Antibacterial Activity

The bacterial strains used for testing antimicrobial activity were *Fusobacterium nucleatum* ATCC25586, *Porphyromonas gingivalis* ATCC33277, *Streptococcus gordonii* ATCC10558 and *Escherichia coli* ATCC27325. All four bacterial strains were obtained from the German Collection of Microorganisms and Cell Cultures (DSMZ, Braunschweig, Germany). For anaerobic growth, the bacteria were cultivated in AnaeroJars with Anaerogen gas packs by Thermo Fisher Scientific (Waltham, MA, USA). Depending on the strain, different cultivation methods and media were used. *F. nucleatum* was cultivated anaerobically at 37 °C in Brain Heart Infusion (BHI) broth (Carl Roth, Karlsruhe, Germany) supplemented with 1 mg/mL yeast extract (Carl Roth) as a liquid medium or on Columbia blood agar with sheep blood (Thermo Fisher Scientific). *P. gingivalis* was cultivated anaerobically at 37 °C in tryptic soy broth (TSB, Merck, Darmstadt, Germany) supplemented with 1 mg/mL yeast extract, 5 µg/mL hemin and 1 µg/mL menadione (both Sigma-Aldrich, St. Louis, MO, USA). For cultivation on plates, 1% Difco agar (Becton Dickinson, Franklin Lakes, NJ, USA) was added to these media. *S. gordonii* and *E. coli* were both cultivated in tryptic soy broth as a liquid medium or TSB with 1% Difco agar as a solid medium under aerobic growth conditions at 37 °C.

The minimal inhibitory concentrations for metronidazole, amoxicillin, doxycycline and rifampicin were determined using Sensi-Discs antibiotic resistance test platelets by Becton Dickinson (Franklin Lakes, NJ, USA). Therefore, liquid cultures of the respective strains were generated, as specified above. These cultures were diluted at 600 nm to an optical density of approximately 0.1 and spread on agar plates, as specified above. Then, the platelets were soaked with different ascending concentrations of the respective antibiotic (between 0.04 and 2000 µg/mL) and positioned on agar plates spread with the respective bacterial strains, as described above. The plates were then incubated aerobically for 24 h (*S. gordonii* and *E. coli*) or anaerobically for 3 to 5 days (*F. nucleatum* and *P. gingivalis*). Finally, the zone of growth inhibition around the platelets was determined. The lowest concentration that resulted in a visible growth inhibition zone was defined as the minimal inhibitory concentration.

The antimicrobial activity of eluates from PCL doped with the different antibiotics were also tested using Sensi-Disc antibiotic resistance test platelets. The platelets were soaked with the respective eluates and then positioned on agar plates spread with the respective bacterial strain, as described above. Then, the plates were incubated for 24 h under aerobic growth conditions (*S. gordonii* and *E. coli*) or anaerobically for 3 to 5 days (*F. nucleatum* and *P. gingivalis*). Finally, it was recorded whether a zone of growth inhibition could be detected surrounding the platelets, and if yes, the diameter of the inhibition zone was measured. The absence of a visible inhibition zone was interpreted as a lack of antimicrobial activity.

### 2.5. Analysis of Cytotoxicity

Immortalised human aortic smooth muscle cells (#P10456-IM, Innoprot) were used. Specifically, 6000 cells were seeded in collagen-coated white 96-well plates in 50 µL SmGM-2 Smooth Muscle Cell Growth (CC-3182, Lonza, Basel, Switzerland) without the addition of antibiotics. After 24 h, 50 µL SmGM-2 with the appropriate antibiotics was added for a further 24 h and cell viability was determined using the RealTime-Glo MT Cell Viability Assay (G9711, Promega, Walldorf, Germany). Luminescence was recorded with a Varioscan LUX Multiplate Reader. The data were normalised to the PCL control (=1).

### 2.6. Statistics

All measurements were performed at least in triplicate and the results are expressed as means ± standard deviation. A one-way or two-way analysis of variance (ANOVA) and a Bonferroni post hoc test were applied for statistical analysis where applicable, and *p* values < 0.05 were considered significant and indicated by an asterisk. To avoid a type II error, the statistical analyses were conducted only across groups and only in selected cases. The cytotoxicity data were analysed using a Kruskal–Wallis test and a Dunn comparison test. The data are presented as scatter dot plots with their median and range.

## 3. Results

### 3.1. Production of PCL-Based DDS

**Figure 1:**
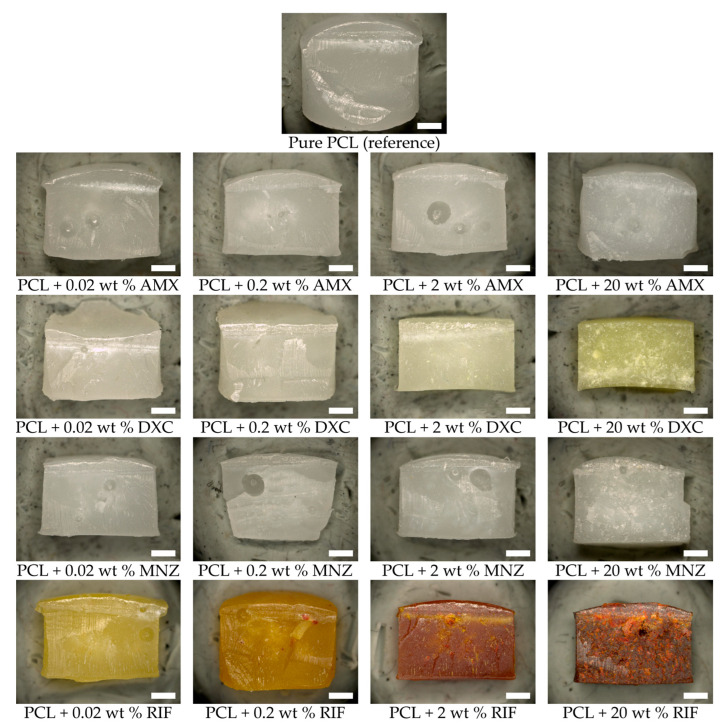
Light microscope images of the PCL-based DDS. Scale bar equals 1 mm.

The antibiotics were successfully incorporated into the polymer matrix by stirring them into the molten PCL. The microscopic images obtained using a light microscope demonstrate a homogeneous distribution of the active ingredients throughout the polymer matrix (Figure 1). In small quantities, the antibiotics are fully soluble in the polymer. As the concentration increases, undissolved particles remain, which are distributed homogeneously in the matrix without forming agglomerates. In certain instances, the introduction of the antibiotics resulted in a change in colour. For instance, rifampicin, which is inherently red, resulted in a yellow colouration at low concentrations and an orange or red colouration at higher concentrations. The addition of doxycycline resulted in a yellowish discolouration of the matrix at high concentrations, while amoxicillin led to an increased opacity of the milky-white matrix. An increase in viscosity was observed as the proportion of the active ingredient in the molten mass increased. The addition of the antibiotics, even in high concentrations, had no negative effect on the moulding process or the dimensional stability of the test specimens.

### 3.2. In Vitro Release of Antibiotics from DDS

**Figure 2:**
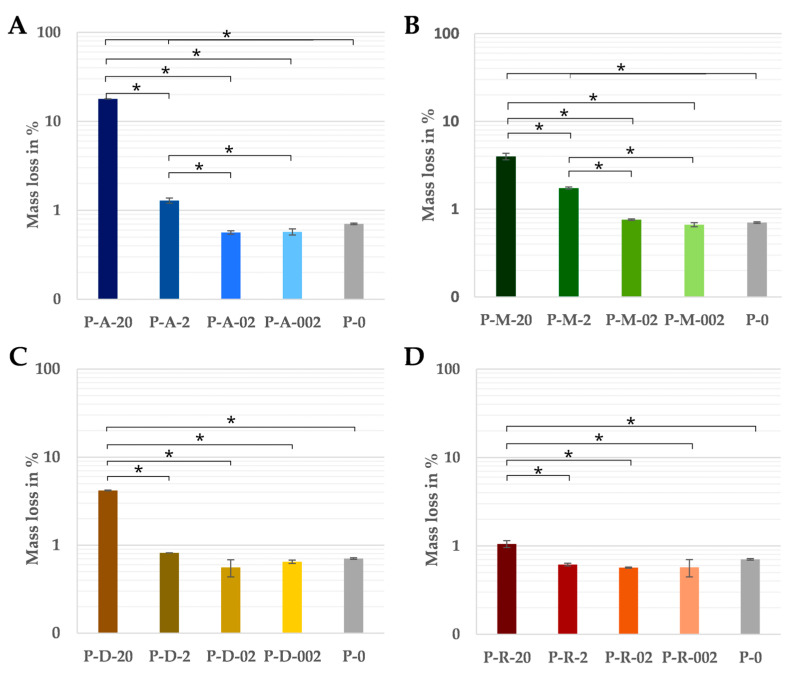
The percentage of mass lost in relation to the initial weight over 14 days of pure PCL and the PCL-based DDS with amoxicillin (**A**), doxycycline (**B**), metronidazole (**C**) and rifampicin (**D**) in PBS at 37 °C (n = 3). Significant differences are indicated by asterisks (*p* < 0.05).

The dry masses of the test specimens before and after incubation showed no significant differences compared to the pure PCL at the two lower concentrations of 0.02 and 0.2 wt%, regardless of the antibiotic. As the absolute amount of antibiotic (0.0142 mg or 0.142 mg) of these samples was within the tolerance of the balance, even the complete dissolution of the antibiotic could not be detected by the change in mass. The greatest mass losses occurred in samples with a content of 20 wt% of the antibiotic (Figure 2). There were also clear differences between the various antibiotics. Of these, the samples with amoxicillin lost the most weight at 17.9% (Figure 2A), the samples loaded with metronidazole (Figure 2B) and doxycycline (Figure 2C)were in the middle range (approximately 4.0%) and the rifampicin variant showed the lowest mass loss at 1.1% (Figure 2D).

**Figure 3:**
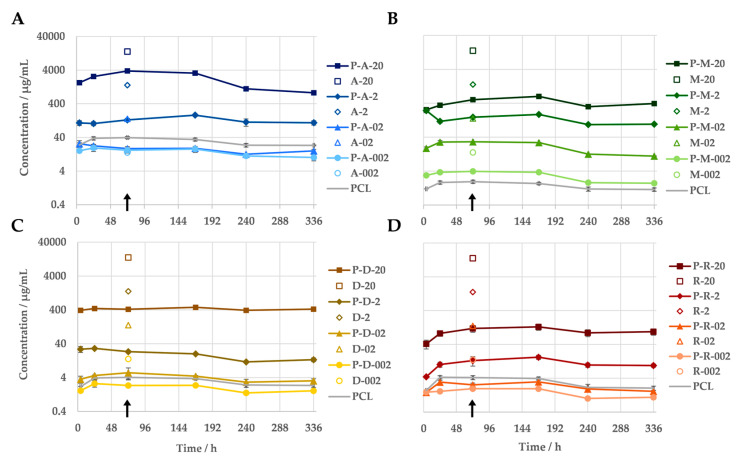
In vitro release profiles of PCL-based DDSs with amoxicillin (**A**), doxycycline (**B**), metronidazole (**C**) and rifampicin (**D**) in PBS (pH = 7.4) at 37 °C, measured via UV/Vis spectroscopy (n = 3). As a reference, the measured antibiotic equivalent of the pure PCL is given. The non-filled markers indicate the antibiotic concentrations that the pure antibiotics caused when they were simply added into 1 mL of medium, according to their mass fraction in the DDS. The difference in the respective antibiotics concentration (from DDS and the plain antibiotic) at the same mass equivalents reflects the sustained release due to the DDS. The samples utilised for the agar diffusion assay, along with the assays for cellular viability, are indicated by an arrow.

The measurement of the antibiotic concentrations in the eluates of the immersion test indicated a correlation between the amount of antibiotic released and the antibiotic concentration in the sample. Regardless of the antibiotic, all PCL-based DDSs released more antibiotic the higher their drug content was (Figure 3). When loaded with 20 wt% antibiotics, between 93% (amoxicillin) and 4% (rifampicin) of the antibiotic was released. All test specimens demonstrated the continuous release of the antibiotic into the medium over the entire 14-day test period. No evidence of a burst release was observed for the PCL-based DDSs under investigation. Nevertheless, the antibiotic was released at a faster rate at the beginning of the incubation during the release measurement. Within the initial 24 h period, a greater quantity of the antibiotic was released from the majority of the samples compared to the subsequent seven days. The release kinetics of the antibiotics differed depending on their specific characteristics, including their concentration. The majority of variants exhibited an increasing release of their antibiotic over the course of the first week. In contrast, the two variants with higher doxycycline concentrations (P-D-20 and P-D-2) and the low-concentration amoxicillin samples (P-A-02 and P-A-002) demonstrated a consistent or declining release from the outset. Furthermore, the quantities released differed considerably between the various antibiotics. The release of the antibiotics gradually decreased as follows: amoxicillin > metronidazole > doxycycline > rifampicin.

The antibiotic equivalents of the pure PCL samples demonstrate an absorption in the same wavelength ranges as the active substances used. With the exception of the metronidazole variants, the apparent concentration of antibiotic in the eluate of the pure PCL exceeds the actual amount of antibiotics in the eluates of the samples with 0.2 and/or 0.02 wt% active substance. The scan of the PCL dissolved in acetonitrile reveals that the material absorbs primarily in the 230–300 nm range.

The release kinetics of the antibiotics from the PCL-based DDSs with 20 wt% drugs were fitted using various models (zero order, first order, Higuchi, Peppa–Sahlin). The best agreement was achieved with the Peppa–Sahlin model (Table 2). As a further development of the frequently used Ritger–Peppas model, it also offers the possibility of estimating the influence of the two release mechanisms, diffusion and relaxation, by means of separate terms [21]. With an R/F ratio consistently below 1 for all samples, diffusion was thus determined as the decisive mechanism.

### 3.3. Efficient Antibacterial Activity of PCL-Based DDS

The released antibiotics from the PCL were then tested for their antimicrobial activity. Therefore, four different strains were used, the two obligate anaerobic Gram-negative species *Porphyromonas gingivalis* and *Fusobacterium nucleatum*, the aerobic Gram-negative species *Escherichia coli* and the Gram-positive aerobic species *Streptococcus gordonii*. First, minimal inhibitory concentrations were determined for the four species towards the employed antibiotics. As expected, *E. coli* and *S. gordonii* were resistant towards metronidazole since this antibiotic is specific towards anaerobic species. However, *P. gingivalis* and *F. nucleatum* were sensitive towards the metronidazole since they were cultured under anaerobic growth conditions. All four tested species were sensitive towards amoxicillin, doxycycline and rifampicin to different degrees (Table 3).

Then, eluates of the antibiotic-doped PCL were tested for their antimicrobial activity by agar diffusion tests. Here, test platelets were loaded with the eluates and placed on agar plates containing a layer of the respective microbial species. As expected, eluates from the pure PCL did not result in any zone of inhibition, showing that PCL by itself has no antimicrobial activity. Also, as expected, eluates from the PCL doped with metronidazole resulted in no zone of inhibition with *S. gordonii* or *E. coli* since these species are resistant to metronidazole. However, eluates from the PCL doped with metronidazole resulted in a large zone of inhibition with *P. gingivalis* and *F. nucleatum*. This was expected since the concentration of metronidazole in the eluates was far higher than the minimal inhibitory concentration of metronidazole towards these species. Also, eluates from the PCL doped with amoxicillin, doxycycline and rifampicin led to clearly detectable zones of inhibition for all four tested bacterial species (Table 4). These results verified that the antibiotics remained functional in all cases during the manufacturing processes.

### 3.4. PCL Loaded with Antibiotics Maintained Cell Viability

**Figure 4:**
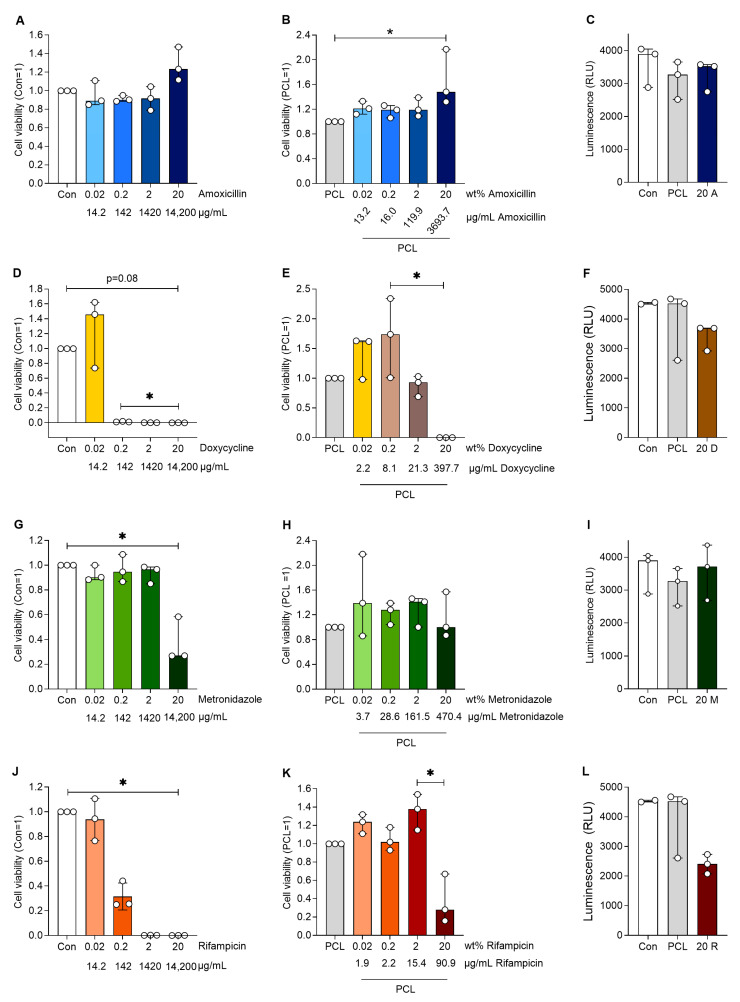
Cell viability of human aortic smooth muscle cells after cultivation with the various plain antibiotics (**A**,**D**,**G**,**J**) at respective antibiotic mass equivalents compared to the antibiotic proportions in the PCL-based DDS. Furthermore, the cell viability of the human aortic smooth muscle cells are shown after cultivation with a medium loaded with antibiotics (released from the PCL-based DDS) with an increasing wt% of different antibiotics (**B**,**E**,**H**,**K**). The luminescence of the human aortic smooth muscle cells (**C**,**F**,**I**,**L**) after cultivation with a preconditioned medium of plain PCL and a medium with 20 wt% mass equivalent antibiotics (14,200 µg/mL) is also shown. Significant differences are indicated by asterisks (*p* < 0.05).

To test the effect of the antibiotic-loaded DDS, human aortic smooth muscle cells were treated with the supernatants of antibiotic-loaded PCL. Cells incubated with antibiotics alone served as controls to elucidate the effect of the slower release from the PCL. Amoxicillin had no negative effect on cell viability and interestingly increased cell viability at the highest loaded concentration compared to PCL alone (*p* < 0.05, Figure 4). Similar effects were observed for amoxicillin alone. Doxycycline almost abolished cell viability from 0.2 wt%, while doxycycline in PCL had no effect on cell viability up to a loading of 20 wt%, which significantly reduced cell viability (*p* < 0.05 20 vs. 0.2 wt%). Rifampicin reduced cell viability at 0.2 wt% and no viable cells were present at higher rifampicin concentrations. The incorporation of rifampicin in PCL reversed this effect, only showing reduced cell viability at 20 wt% (*p* < 0.05 20 vs. 2 wt%). Metronidazole alone reduced cell viability only at the highest concentration (equivalent of 20 wt%), whereas when incorporated in PCL, it had no effect on cell viability at any of the concentrations used.

Human aortic smooth muscle cells were incubated with media (Con), media containing pure antibiotics (amoxicillin, doxycycline, rifampicin and metronidazole) without PCL and PCL loaded with antibiotics. Media without cells served as a blank control. Cell viability was analysed after 24 h of treatment, and the data were normalised to PCL (=1) or cells with the corresponding antibiotics (=1). The table below the following figures shows the wt% values indicating the loading of the corresponding antibiotics in the PCL. The C-release (µg/mL) presents the measured concentrations of the drug in the eluate and the concentrations at which the cells were incubated.

## 4. Discussion

There is a certain need for DDSs for local applications that enable the long-term release of active ingredients by incorporating large quantities of antibiotics that are otherwise cytotoxic when applied directly. Therefore, a DDS based on the hydrophobic polyester PCL with up to 20 wt% antibiotics was produced by mould casting, a melt-based process, enabling the incorporation of high quantities of additives into the PCL, as described in the literature [19,22]. The low melting point of PCL allows the material to be processed at approximately 90 °C. In comparison to numerous other biodegradable synthetic polymers, this temperature is relatively low [23], thereby ensuring a gentle processing of the antibiotics. Microbiological inhibition tests, which encompass a diverse range of both Gram-positive and Gram-negative anaerobic and aerobic pathogens, demonstrated that the antibacterial efficacy of the antibiotics remained unimpaired even after incorporation into PCL at this elevated temperature and a processing time of up to 60 min.

This manufacturing approach enabled the production of stable test specimens with defined proportions of up to 20 wt% antibiotic. A review of the literature reveals that the potential for further development remains, with the possibility of incorporating even larger quantities of the additive [19]. Nevertheless, the quantity of active ingredient achieved in this instance is already comparable with typical single doses for the systemic administration of antibiotics in the form of film-coated tablets. Due to the avoidance of the first-pass effect (prior metabolization of the active substance by the liver), significantly lower doses are required for local application [3]. However, a high loading quantity is recommended to achieve a long-lasting effect. This requires the release of the antibiotics to be slowed down by the matrix material, whereby the DDS can be regarded as a depot from which the active substance is continuously released.

The in vitro experiments demonstrated that the release from PCL is diffusion-driven, with minimal material degradation (<1 wt%) over a period of 14 days. This conclusion is also corroborated by the fit of the Peppa–Sahlin model. This suggests that only a minor fraction of the release can be attributed to relaxation processes in the matrix polymer. The hydrophobic nature of PCL results in minimal water uptake, preventing any significant swelling or rearrangement of the polymer chains. Consequently, the chain relaxation cannot contribute to the release of the active substances. It is also noteworthy that there is a significant difference between the various antibiotics. The varying dissolution and, consequently, the varying release rates of the active substance from the PCL matrix can be attributed to the interplay between the hydrophobic nature of PCL and the distinct chemical properties of the incorporated antibiotics. The hydrophilic antibiotic amoxicillin demonstrated the highest release rate (93%) in comparison to the more lipophilic antibiotics. Rifampicin, being the least hydrophilic, exhibited a release of only 4% during the 14-day period. Doxycycline and metronidazole exhibited an intermediate release. Thus, the primary factor influencing the diffusion rate of additives in the PCL matrix is its water solubility, which has been shown earlier by Schüler et al. [22]. Thus, the required amount of antibiotic can be adjusted by the loading quantity according to its solubility. This pattern emerges from the compatibility between the drug and the hydrophobic PCL matrix, where lipophilic drugs like rifampicin have stronger interactions with the polymer matrix, resulting in slower release rates [24]. The release mechanism, confirmed by the Peppas–Sahlin model with R/F ratios consistently below 1, is predominantly diffusion-controlled [25]. In this system, drug molecules must navigate through the dense hydrophobic polymer chains of PCL, which act as a barrier to diffusion [26]. Hydrophilic drugs, having less affinity for the PCL matrix, demonstrate faster diffusion rates through the polymer network, while hydrophobic drugs remain more strongly bound within the matrix, leading to more sustained release profiles [24]. This mechanism explains the observed release hierarchy, amoxicillin > metronidazole > rifampicin, which correlates directly with their decreasing hydrophilicity.

Given that the active ingredients were not released completely within the 14 d in vitro test, it can be postulated that this process continues beyond the two-week trial period. In certain cases, there is a medical need for an extended treatment period, particularly in instances where there is a possibility of delayed infection, such as VGI. For these cases, our carrier system would be particularly suitable.

The requisite long-term release remains a significant challenge. One factor related to the material that affects diffusion is hydrophilicity. A review of the literature on the degradation of biocompatible polyesters, including PCL and poly(lactic-co-glycolic) (PLGA), indicates that an increase in hydrophobicity and a reduction in the swelling factor, as observed in PCL, result in a slower release of drugs [17]. It has been demonstrated that the combination of a hydrophobic matrix material, such as PCL, and an equally hydrophobic active ingredient, such as rifampicin, is a promising approach. The findings indicate that PCL can serve as a carrier material and that the optimal combination of active ingredient and loading quantity has the potential to adapt the duration of treatment to patient-specific needs.

In addition, the results of the cytotoxicity tests indicate that the loading of antibiotics into a PCL matrix reduced cytotoxicity, indicating a slower release with lower concentrations acting on the smooth muscle cells. This becomes particularly clear when compared to the effect of pure antibiotics on these cells. While amoxicillin had no cytotoxic effect, rifampicin, metronidazole and doxycycline reduced cell viability in a dose-dependent manner. Similar results were obtained in gingival fibroblasts and human osteoblasts [27,28,29]. Our data show that the balance between achieving antimicrobial effects and avoiding cell toxicity is a critical issue, especially during local delivery [29]. DDSs can extend the time of action, improve efficacy and specific targeting, and reduce toxicity [30]. Similar effects were demonstrated in human gingival fibroblasts with PLGA-coated ceramic microparticles as a DDS [27], vancomycin in PCL (Pyramid-Shaped PEG-PCL-PEG Polymeric-Based Model Systems for Site-Specific Drug Delivery of Vancomycin with Enhanced Antibacterial Efficacy) and metronidazole- and amoxicillin-loaded PLGA and PCL nanofibers [17].

## 5. Conclusions

With this work, we were able to show that the PCL-based drug delivery systems were successfully manufactured by mould casting, which allowed us to adjust a wide range of antibiotic loadings of four orders of magnitude. With this DDS, an effective slowdown of drug release while maintaining antibacterial efficacy is possible. Furthermore, the maximum locally released drug concentrations were cell compatible. In conclusion, PCL-based DDSs offer an effective therapeutic window for local antibiotic administration. This provides novel therapeutic options in endoprosthetics, as well as the treatment of periodontitis and vascular graft infection.

## Figures and Tables

**Table 1:** Parameters of UV/Vis-based antibiotic measurement in PBS. Specification of the characteristic wavelengths and the equation of the regression lines for the calibration curves for all four antibiotics (regression coefficient based on triplicate measurements).

Antibiotic	Characteristic Wavelength	Regression Line	Regression Coefficient
Amoxicillin	274 nm	y = 0.9887 x	R^2^ = 0.9998
Doxycycline	346 nm	y = 7.0098 x	R^2^ = 0.9992
Metronidazole	320 nm	y = 16.767 x	R^2^ = 1
Rifampicin	475 nm	y = 4.7681 x	R^2^ = 0.9983

**Table 2:** Correlation coefficients obtained by fitting the antibiotic release data to the zero-order, first-order, Higuchi, and Peppas–Sahlin models.

Sample	Zero Order	First Order	Higuchi	Peppas–Sahlin (m = 0.448)		
	R^2^	R^2^	R^2^	R^2^	K1	K2
P-A-20	0.8484	0.9907	0.9975	1	0.7957	0.0553
P-D-20	0.92	0.9341	0.9949	0.9967	0.1695	0.0006
P-M-20	0.9392	0.9503	0.9996	0.9994	0.1476	0.0025
P-R-20	0.9597	0.9529	0.998	0.9991	0.0297	0.0009

**Table 3:** Minimal inhibitory concentrations for the analysed antibiotics. MICs were determined using disc diffusion tests. Therefore, Sensi-Discs were loaded with ascending concentrations of the respective antibiotics. Agar diffusion tests were then conducted with the indicated bacterial strains. The loaded test platelets were put on agar plates spread with the respective bacterial strain. The zone of inhibition was read out after 24 h (*S. gordonii*, *E. coli*) or 3–5 days (*P. gingivalis*, *F. nucleatum*). This Table shows the lowest concentration where a zone of inhibition could be detected.

	MIC in µg/mL			
Antibiotic	*P. gingivalis*	*F. nucleatum*	*S. gordonii*	*E. coli*
Amoxicillin	5	250	2	100
Doxycycline	5	5	2	50
Metronidazole	10	1	resistant	resistant
Rifampicin	0.5	350	9	300

**Table 4:** Antibiotic activity of the released antibiotics from PCL. Sensi-Disc test platelets were loaded with the eluates released from PCL doped with various antibiotics. Agar diffusion tests were then conducted with the indicated bacterial strains. The loaded test platelets were put on agar plates spread with the respective bacterial strain. The zone of inhibition was read out after 24 h (*S. gordonii*, *E. coli*) or 3–5 days (*P. gingivalis*, *F. nucleatum*). A 0 indicates that no inhibition zone could be detected and >50 mm indicates that the inhibition zone reached the edge of the agar plates.

		Diameter of Inhibiting Zone in mm			
Antibiotic	Concentration in µg/mL	*P. gingivalis*	*F. nucleatum*	*S. gordonii*	*E. coli*
PCL	-	0	0	0	0
PCL + amoxicillin	3762.7	>50	46	26	7
PCL + doxycycline	397.3	>50	>50	30	16
PCL + metronidazole	495.1	>50	46	0	0
PCL + rifampicin	120.1	>50	34	26	0

## Data Availability

The original data presented in the study are openly available in OPARA at https://doi.org/10.25532/OPARA-853.

## Supplementary Materials

The following supporting information can be downloaded at https://www.mdpi.com/article/10.3390/jfb16050182/s1, Figure S1: Low-concentration solutions were prepared for the purpose of determining the characteristic extinction maxima of the four antibiotics. Each 50 µL sample volume was analyzed in UV-Star^®^ Microplates (Greiner Bio-One, Frickenhausen, Germany) using the Infinite 200Pro (TECAN, Männedorf, Switzerland) in a wavelength range of 230–700 nm. The same procedure was followed for PCL dissolved in acetonitrile in a quartz cuvette (Hellma, Jena, Germany). The characteristic extinction maxima determined in this way were used for further measurements; Figure S2: The antibiotic concentrations present in the samples are determined from the measured absorbance values. For this purpose, three calibration series through the zero point (R^2^ ≥ 0.998) were created for all antibiotics in PBS, with the absorbance values of the pure PBS subtracted as blanks. A mean regression line was determined from these three, which was then used to convert the measured absorbance values of the samples into antibiotic concentrations.

## References

[1] Leekha S., Terrell C.L., Edson R.S. (2011). General principles of antimicrobial therapy. Mayo Clin. Proc..

[2] Sullivan Å., Edlund C., Nord C.E. (2001). Effect of antimicrobial agents on the ecological balance of human microflora. Lancet Infect. Dis..

[3] Jain K.K. (2020). An Overview of Drug Delivery Systems. Methods Mol. Biol..

[4] Budală D.G., Luchian I., Tatarciuc M., Butnaru O., Armencia A.O., Virvescu D.I., Scutariu M.M., Rusu D. (2023). Are Local Drug Delivery Systems a Challenge in Clinical Periodontology?. J. Clin. Med..

[5] Cara A., Ferry T., Laurent F., Josse J. (2022). Prophylactic Antibiofilm Activity of Antibiotic-Loaded Bone Cements against Gram-Negative Bacteria. Antibiotics.

[6] Mufty H., Bergh M.V.D., Meuris B., Metsemakers W.-J., Fourneau I. (2022). Clinical Studies Reporting on Vascular Graft Coatings for the Prevention of Aortic Graft Infection: A Systematic Review and Meta-Analysis. Eur. J. Vasc. Endovasc. Surg..

[7] Mufty H., Eynde J.V.D., Meuris B., Metsemakers W.-J., Van Wijngaerden E., Vandendriessche T., Steenackers H.P., Fourneau I. (2021). Pre-clinical in vivo Models of Vascular Graft Coating in the Prevention of Vascular Graft Infection: A Systematic Review. Eur. J. Vasc. Endovasc. Surg..

[8] Chakfé N., Diener H., Lejay A., Assadian O., Berard X., Caillon J., Fourneau I., Glaudemans A.W., Koncar I., Lindholt J. (2020). Editor’s Choice—European Society for Vascular Surgery (ESVS) 2020 Clinical Practice Guidelines on the Management of Vascular Graft and Endograft Infections. Eur. J. Vasc. Endovasc. Surg..

[9] Lovering A., MacGowan A. (1999). A Comparative study of the rifampicin binding and elution characteristics for collagen- and albumin-sealed vascular grafts. Eur. J. Vasc. Endovasc. Surg..

[10] Aboshady I., Raad I., Vela D., Hassan M., Aboshady Y., Safi H.J., Buja L.M., Khalil K.G. (2015). Prevention of perioperative vascular prosthetic infection with a novel triple antimicrobial-bonded arterial graft. J. Vasc. Surg..

[11] Matsuura S., Takayama T., Oyama T.G., Oyama K., Taguchi M., Endo T., Akai T., Isaji T., Hoshina K. (2021). A Radiation-Crosslinked Gelatin Hydrogel That Promotes Tissue Incorporation of an Expanded Polytetrafluoroethylene Vascular Graft in Rats. Biomolecules.

[12] Yap C., Mieremet A., de Vries C.J., Micha D., de Waard V. (2021). Six Shades of Vascular Smooth Muscle Cells Illuminated by KLF4 (Krüppel-Like Factor 4). Arterioscler. Thromb. Vasc. Biol..

[13] Johansen M.I., Rahbek S.J., Jensen-Fangel S., Minero G.A.S., Jensen L.K., Larsen O.H., Erikstrup L.T., Seefeldt A.M., Østergaard L., Meyer R.L. (2023). Fibrinolytic and antibiotic treatment of prosthetic vascular graft infections in a novel rat model. PLoS ONE.

[14] Rosalia M., Grisoli P., Dorati R., Chiesa E., Pisani S., Bruni G., Genta I., Conti B. (2023). Influence of Electrospun Fibre Secondary Morphology on Antibiotic Release Kinetic and Its Impact on Antimicrobic Efficacy. Int. J. Mol. Sci..

[15] Holländer J., Genina N., Jukarainen H., Khajeheian M., Rosling A., Mäkilä E., Sandler N. (2016). Three-Dimensional Printed PCL-Based Implantable Prototypes of Medical Devices for Controlled Drug Delivery. J. Pharm. Sci..

[16] Osehontue Uroro E., Bright R., Yang Quek J., Vasilev K. (2023). Lipase-Responsive Rifampicin-Based Biodegradable PCL Nanocarrier for Antibacterial Treatment. ChemNanoMat.

[17] Mirzaeei S., Mansurian M., Asare-Addo K., Nokhodchi A. (2021). Metronidazole- and Amoxicillin-Loaded PLGA and PCL Nanofibers as Potential Drug Delivery Systems for the Treatment of Periodontitis: In Vitro and In Vivo Evaluations. Biomedicines.

[18] Woodruff M.A., Hutmacher D.W. (2010). The return of a forgotten polymer—Polycaprolactone in the 21st century. Prog. Polym. Sci..

[19] Flath T. (2020). Entwicklung eines Doppelschneckenextruder-Dosierkopfes für den 3D-Druck und dessen Potenzial am Beispiel von Knochenersatzwerkstoffen. Ph.D. Dissertation.

[20] Liu Y., Chen X., Liu Y., Gao Y., Liu P. (2022). Electrospun Coaxial Fibers to Optimize the Release of Poorly Water-Soluble Drug. Polymers.

[21] Bruschi M. (2015). Strategies to Modify the Drug Release from Pharmaceutical Systems: Mathematical Models of Drug Release.

[22] Schüler T., Guder C., Alt F., Lorenz K., Sterzenbach T., Hannig C., Wiesmann H.-P., Kruppke B. (2024). Degradable polycaprolactone/buffer composites as pH regulating carrier materials for drug delivery and 3D printed biomaterials. Materialia.

[23] Chesterman J., Zhang Z., Ortiz O., Goyal R., Kohn J. (2020). Principles of Tissue Engineering.

[24] Fu Y., Kao W.J. (2010). Drug release kinetics and transport mechanisms of non-degradable and degradable polymeric delivery systems. Expert Opin. Drug Deliv..

[25] Talevi A., Ruiz M.E. (2022). Korsmeyer-Peppas, Peppas-Sahlin, and Brazel-Peppas: Models of Drug Release. The ADME Encyclopedia.

[26] Gurny R., Doelker E., Peppas N. (1982). Modelling of sustained release of water-soluble drugs from porous, hydrophobic polymers. Biomaterials.

[27] Ferreira M.B., Myiagi S., Nogales C.G., Campos M.S., Lage-Marques J.L. (2010). Time- and concentration-dependent cytotoxicity of antibiotics used in endodontic therapy. J. Appl. Oral Sci..

[28] Duewelhenke N., Krut O., Eysel P. (2007). Influence on mitochondria and cytotoxicity of different antibiotics administered in high concentrations on primary human osteoblasts and cell lines. Antimicrob. Agents Chemother..

[29] Rathbone C.R., Cross J.D., Brown K.V., Murray C.K., Wenke J.C. (2011). Effect of various concentrations of antibiotics on osteogenic cell viability and activity. J. Orthop. Res..

[30] Zong T.-X., Silveira A.P., Morais J.A.V., Sampaio M.C., Muehlmann L.A., Zhang J., Jiang C.-S., Liu S.-K. (2022). Recent Advances in Antimicrobial Nano-Drug Delivery Systems. Nanomaterials.

