# Supplementary material for: Versatile Polycaprolactone-Based Drug Delivery System with Enhanced Cytocompatibility and Antibacterial Activity
Source: J Funct Biomater. 2025 May 15;16(5):182. doi: 10.3390/jfb16050182 (PMC12111853; doi:10.3390/jfb16050182)
Supplement: Supplementary file 1 [file jfb-16-00182-s001.zip › jfb-3584621-supplementary.pdf]

Journal of
Functional Biomaterials

Article

Versatile polycaprolactone-based Drug Delivery System with enhanced cytocompatibility and antibacterial activity

Celine Guder [1], Anja Hofmann[2], Therese Schüler [1], Torsten Sterzenbach [3], Hans-Peter Wiesmann [1], Katrin Lorenz [3], Christian Hannig [3], Christian Reeps[2] and Benjamin Kruppke [1*]

[1] Max Bergmann Center of Biomaterials and Institute of Materials Science, Technische Universität Dresden, 01069 Dresden, Germany
[2] Division of Vascular and Endovascular Surgery, Department of Visceral, Thoracic and Vascular Surgery, Medical Faculty and University Hospital Carl Gustav Carus, Technische Universität Dresden, 01307, Dresden, Germany
[3] Policlinic of Operative Dentistry, Periodontology and Pediatric Dentristy, Medical Faculty Carl Gustav Carus, Technische Universität Dresden, 01307 Dresden, Germany
* Correspondence: Tel.: +49 351 463 42762; fax: +49 351 463 39401; E-mail address: Benjamin.Kruppke@tu-dresden.de

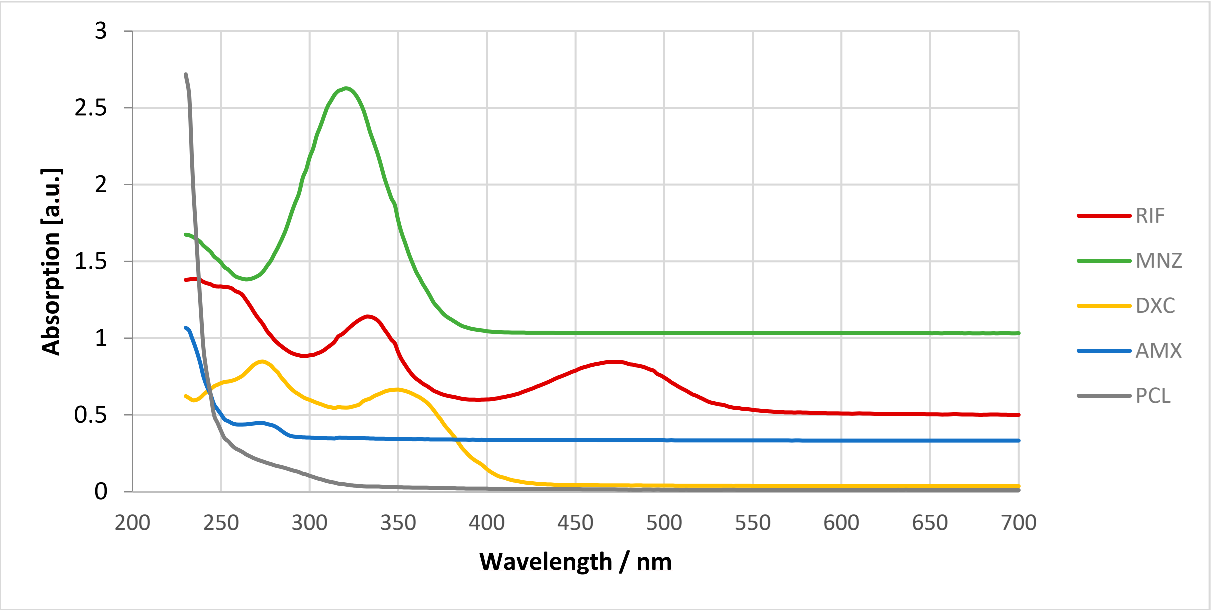

Figure S1. Low-concentration solutions were prepared for the purpose of determining the characteristic extinction maxima of the four antibiotics. Each 50 µL sample volume was analyzed in UV-Star® Microplates (Greiner Bio-One) using the Infinite 200Pro (TECAN) in a wavelength range of 230–700 nm. The same procedure was followed for PCL dissolved in acetonitrile in a quartz cuvette (Hellma, Germany). The characteristic extinction maxima determined in this way were used for further measurements.

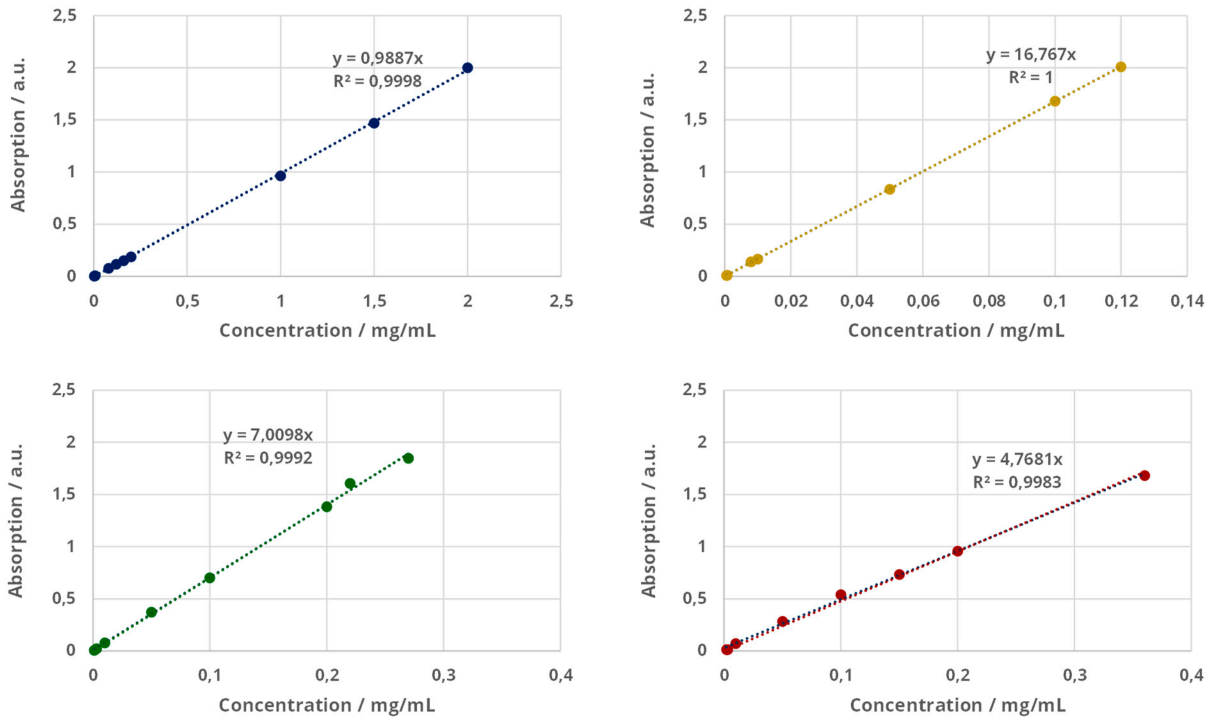

Figure S2. The antibiotic concentrations present in the samples are determined from the measured absorbance values. For this purpose, three calibration series through the zero point ($R^2 \geq 0.998$) were created for all antibiotics in PBS, with the absorbance values of the pure PBS subtracted as blanks. A mean regression line was determined from these three, which was then used to convert the measured absorbance values of the samples into antibiotic concentrations.
